# Managing self-harm in young people presenting to the emergency department and challenges in navigating the national guidelines

**DOI:** 10.1192/bjb.2023.24

**Published:** 2023-08

**Authors:** Naashoma Pereira Carvalho, Kim Pierson, Hadi Shaker-Naeeni, Esther Sabel

**Affiliations:** 1 North Central London Higher Training Programme, London, UK; 2 Hertfordshire Partnership University NHS Foundation Trust, Hatfield, UK

**Keywords:** Epidemiology, community mental health teams, self-harm, NICE guidelines, RCPsych guidelines

## Abstract

Suicide is the most common cause of death for young people in the UK and around 50% of completed suicides in young people have previous self-harm as a theme. Hence, robust management of young people presenting with self-harm to the emergency department is crucial. Guidelines published by the National Institute for Health and Care Excellence (NICE) and the Royal College of Psychiatrists advise an overnight admission for under-16s with self-harm, which is a challenge during winter pressures or bed shortages. In this editorial we discuss the difficulties faced when navigating NICE 2004 guidance documents with the realities of the coalface and consider the prospects for current practice and the future with the NICE 2022 guidance.

The UK has one of the highest rates of self-harm in Europe (400 episodes per 100 000 population). Self-harm increases the likelihood of eventually dying by suicide by 50- to 100-fold compared with the rest of the population.^1^

The 2017 National Confidential Inquiry into Suicide and Homicide by People with Mental Illness reported that 50% of the completed suicides in young people had previous self-harm as a theme.^2^ Psychosocial assessment appears to be beneficial in reducing the risk of self-harm repetition.^3^

## Management guidance to date

According to our interpretation, the ethos of guidelines published by the National Institute for Health and Care Excellence (NICE) in 2004^4^ and by the Royal College of Psychiatrists (RCPsych) in 2014,^1^ in suggesting overnight admission for under 16-year olds, served to offer respite, a ‘cooling off’ period and an opportunity to ‘wrap the network around the child’.^5^ It is an opportunity to do a comprehensive and longitudinal biopsychosocial assessment of their presentation and risk, and crucially to effect change by intervening.

For 16- to 17-year olds the guidelines had differed. The ‘blanket’ recommendation of an acute hospital admission was not insisted on in all cases, as it was for the under-16s. In the NICE 2004 guidelines, young people were defined as those under 16 and special considerations for 16- to 17-year olds were not discussed. However, the RCPsych 2014 guidelines addressed this gap about what to do for 16- to 17-year olds and cautioned against discharge without a very thorough biopsychosocial assessment in this even more risky age group, stating: ‘if in any doubt [about safety or about quality of information, then acute hospital], admission should follow'.^1^

## Adaptations to practice since COVID-19

We are working in an increasingly unusual clinical context, when it comes to the management of young people presenting to the emergency department with self-harm. Year on year, even prior to the COVID-19 pandemic, such presentations have risen exponentially.^2^ Policy documents such as Future in Mind^6^ and the Crisis Care Concordat^7^ have highlighted the need for, and even requested, swift and expert assessments by clinicians from child and adolescent mental health services (CAMHS) to take place for crisis presentations within 4 h, thus meeting the same rapid standards set for adults.

At the start of COVID-19, our local CAMHS crisis team changed their operational hours from 9 a.m.–9 p.m. to a 24/7 service, in accordance with an urgent national request by NHS England. This was applicable to all similar services across England and this service change has stayed in place. The NICE 2022 guidelines on self-harm have effectively endorsed it, advocating psychosocial assessment ‘at the earliest opportunity’.^8^

Acute hospital stakeholders may believe that rapid assessment should equate to rapid discharge. Consequently, there has recently been a potential paradox between policy and practice, with an expectation of a rapid assessment but still offering overnight admission when indicated, when following the NICE 2004 guidance. Since 24/7 services started, but prior to the release of the NICE 2022 guidance, we aimed to balance the instruction for rapid assessment with the wish to follow NICE 2004 guidance on overnight admission, by going back to basics and considering what the purpose, and essentially the ethos, of the NICE guidelines as originally created might be. According to our interpretation of the ethos, the NICE 2022 guidelines have broadly stuck by it (in advocating admission where it is not safe to discharge for various reasons), except they do not explicitly talk about offering interventions in the setting of an acute hospital but just mention offering a psychosocial assessment.

With many services for both young people and adults now running 24/7 in the wake of COVID-19, it was worth considering questions like ‘What is the definition of overnight?’ when considering how and whether to offer the ‘overnight admission’. However, the new NICE 2022 guidelines have potentially improved clarity on the question of overnight admissions and duration of admission.

When a child presents with self-harm, one must consider that this represents a desperate communication which above all must be heard and understood. These communications are fraught with distress and danger. There may be complex family dynamics and/or safeguarding disclosures, which need adequate time to unfold and must be handled sensitively. Ultimately, we need to develop a biopsychosocial formulation to facilitate a deep understanding, within the ‘network’ and within the family, of why this child has presented in this way at this time. The implicit question that follows is ‘What must be done to enable them to safely go home?’ – if home is indeed the suggested course of action.

When working with young people, it is important to collaborate with the agencies involved or those that need to become involved – hence the idea of ‘wrapping the network around the child’.^5^ However, for presentations that occur at night, the network, including professionals already involved, are asleep. The later in the day that a young person presents, the less chance of meaningful collaboration with that network. Considering the factors, such as COVID-19 and pressures on acute hospitals, it makes sense that the service response is rapid, as requested by recent policy. However, as per NICE guidelines, the response needs also to be thoughtful, allowing for longitudinal assessment and engagement with the network, for a moment of respite and for change to occur. Given that networks are typically unavailable out of hours, it is much more challenging to effect a change at night via an external network.

Hawton et al reported that presentations of self-harm in young people peak out of hours.^9^ Nadkarni et al also showed that many children and young people attending emergency departments with self-harm present alone or with non-family members.^10^ This further complicates the process of assessing and gathering information about services they are already under. If they present in the morning rather than at night, it is easier to complete a comprehensive biopsychosocial risk assessment and formulation, which allows events to unfold over several hours, as befits the gravity of the presentation, and then ‘wrap the network around the child’, achieving change. We would then propose that discharge be considered concordant with the original aims and ethos of NICE 2004 recommendations, but more streamlined, allowing a discharge ahead of the night. We propose that if there has been a positive shift in mental state and an active network has been mobilised around the young person then a considered daytime discharge could be in keeping with the ethos of the NICE 2004 and RCPsych guidelines and now also the new NICE 2022 guidelines, despite the young person potentially not having stayed overnight.

## The horizon ahead

The NICE 2022 guidelines^8^ recommend, in line with the RCPsych guidance,^1^ that admission to a general hospital is considered:

- if there are concerns about safety (e.g. if the person is at a risk of violence, abuse or exploitation) or if safeguarding planning needs to be completed and a psychiatric admission is not indicated – however, we are surprised that concern about imminent risk of suicide is not explicitly stated as an example of a safety concern
- if the person is unable to engage, for example because of distress or intoxication,

and that

1. they should have a full psychosocial assessment as well as care planning, discharge planning and aftercare organisation – we applaud the changes for 16- to 17-years-olds ensuring that this is to a ward that can meet the needs of young people,

and that

1. there should be no delay in carrying out a psychosocial assessment or offering mental health treatment if the person is admitted to hospital or needs treatment for physical injuries. Additionally, the NICE 2022 guidance champions young people's rights effectively by asking for age-appropriate liaison services within specialist CAMHS teams, 24/7.

It is striking that, as resonates with our current practice described above, what is being suggested in the NICE 2022 guidelines is an approach that is both more pragmatic and more nuanced yet still broadly loyal, in our opinion, to the ethos that underpins the NICE 2004 guidelines. Essentially, admissions are still suggested under certain circumstances that would cause clinical alarm and these circumstances are broad enough to justify admission in many cases but also leave it up to the knowledge, experience and concern of the practitioner on a case-by-case basis rather than adopting a universal rule.

We concur that the current guideline direction appears to be veering towards both a practical and a thoughtful stance. However, we would like to highlight some small considerations for caution.

In its rationale for no longer suggesting a ‘blanket’ admission for all young people following an emergency self-harm presentation, the NICE 2022 guidelines committee concluded, based on available evidence (Waterhouse & Platt's 1990 study^11^) and on its ‘experience and knowledge’, that for people of all ages with such presentations admission to hospital carries a greater risk of distress than benefit. However, the committee also acknowledged the value of time to recover. Bearing in mind these young people are already severely distressed and may be at risk of death by suicide, suggesting that the admission may ‘cause distress’ is possibly missing the point of the admission.

The evidence (Waterhouse & Platt^11^) on which this decision is based concludes that there are no short-term or long-term differences in repeated self-harm regardless of whether people are offered an admission or discharged home. Crucially, this study is from 1990, i.e. before the 2004 NICE guidelines, and one would argue as to why it was not deemed relevant when making the 2004 admission recommendation. Also, the patient group were all over 16, so although the NICE committee acknowledges this study, it rates the evidence quality as ‘moderate’ and also names ‘serious imprecision for the evidence regarding this outcome’ in young people, for example those under 16. This study also excluded people who ‘did not require psychiatric care’, but what does ‘did not require psychiatric care’ even mean regarding the relevance of hospital admission for young people?

It is also worth considering whether parity principles have been applied to this consideration about distress when discussing this matter as a consideration in avoiding admission. For example, one of us, a paediatrician, is well versed in navigating hospital admissions for life-threatening physical health conditions such as severe sepsis, where distress caused by admission would never be considered a barrier. The distress is something to be thought about and reduced where possible through use of distraction techniques, play-team involvement, activities to keep the patient occupied and environmental adjustments to the ward. The NICE 2022 guidelines recommend admitting young people to an age-appropriate ward, which would reduce this distress. However, the distress caused by the admission itself is not a reason to discharge an unwell, unstable patient. The astute reader might wonder whether a ‘kind suggestion’ about avoiding distress by avoiding hospital admission might be another example of covert stigma, in that these young people might be not only distressed but also distressing, i.e. to staff by their very presence or a difficult countertransference being acted out – and that this occurs in a climate of limited bed availability and resources, where demand and capacity are often woefully mismatched.

The NICE guidelines 2022 additionally suggest that there is no evidence but, based on their experience and knowledge, therapeutic risk-taking has benefits. However, given that there is no evidence, they could not be more specific about when therapeutic risk-taking should be considered. But the guidelines mention that therapeutic risk-taking should only be used after a psychosocial assessment.

Although this update of the NICE guidance regarding overnight admission following self-harm was no ‘fly-by-night’ undertaking, caution about how we practise and how we apply it is advised, because professional anxieties can be and should be justifiably high and, in terms of available evidence, ‘the night is still young’.

## About the authors

**Naashoma Pereira Carvalho**, MBBS, MRCPsych, is a psychiatry registrar in general adult psychiatry on the North Central London Higher Training Programme, London, UK. **Kim Pierson**, MBBS, MRCPCH, PGCert Health Psychology, BSc, is a paediatric registrar in child mental health with Hertfordshire Partnership University NHS Foundation Trust, Hatfield, UK. **Hadi Shaker-Naeeni**, MD, MRCPsych, is a child and adolescent consultant psychiatrist and medical lead for quality and safety of CAMHS, Hertfordshire University Partnership NHS Foundation Trust, Hatfield, UK. **Esther Sabel**, MBChb(Eur), MRCPsych, MA Clin Ed, is a consultant child and adolescent psychiatrist in a CAMHS crisis assessment and treatment team, Hertfordshire University Partnership NHS Foundation Trust, Hatfield, UK.

## Data Availability

Data availability is not applicable to this article as no new data were created or analysed in this study.
